# qPMS7: A Fast Algorithm for Finding (*ℓ*, *d*)-Motifs in DNA and Protein Sequences

**DOI:** 10.1371/journal.pone.0041425

**Published:** 2012-07-24

**Authors:** Hieu Dinh, Sanguthevar Rajasekaran, Jaime Davila

**Affiliations:** 1 Department of Computer Science and Engineering, University of Connecticut, Storrs, Connecticut, United States of America; 2 Division of Biomedical Statistics and Informatics, Mayo Clinic, Rochester, Minnesota, United States of America; Dana-Farber Cancer Institute, United States of America

## Abstract

Detection of rare events happening in a set of DNA/protein sequences could lead to new biological discoveries. One kind of such rare events is the presence of patterns called motifs in DNA/protein sequences. Finding motifs is a challenging problem since the general version of motif search has been proven to be intractable. Motifs discovery is an important problem in biology. For example, it is useful in the detection of transcription factor binding sites and transcriptional regulatory elements that are very crucial in understanding gene function, human disease, drug design, etc. Many versions of the motif search problem have been proposed in the literature. One such is the 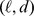 *-motif search* (or *Planted Motif Search (PMS)*). A generalized version of the PMS problem, namely, *Quorum Planted Motif Search (qPMS)*, is shown to accurately model motifs in real data. However, solving the qPMS problem is an extremely difficult task because a special case of it, the PMS Problem, is already NP-hard, which means that any algorithm solving it can be expected to take exponential time in the worse case scenario. In this paper, we propose a novel algorithm named qPMS7 that tackles the qPMS problem on real data as well as challenging instances. Experimental results show that our Algorithm qPMS7 is on an average 5 times faster than the state-of-art algorithm. The executable program of Algorithm qPMS7 is freely available on the web at http://pms.engr.uconn.edu/downloads/qPMS7.zip. Our online motif discovery tools that use Algorithm qPMS7 are freely available at http://pms.engr.uconn.edu or http://motifsearch.com.

## Introduction

Detection of rare events happening in a set of DNA/protein sequences often provides the main clue leading to new biological discoveries. One kind of such rare events is the presence of patterns called motifs. For example, regulatory regions in a genome such as promoters, enhancers, locus control regions, etc., contain motifs that control many biological processes such as gene expression (see [1]). Basically, proteins known as transcription factors regulate the expression of a gene by binding to locations of motifs in regulatory regions. For instance, transcription factors such as TFIID, TFIIA and TFIIB usually bind to sequence 5′-TATAAA-3′ in the promoter region of a gene in order to initiate its transcription. Such motifs and their locations in regulatory regions, i.e., binding sites, are important and helpful to decipher the regulatory mechanism of gene expression, which is very sophisticated. As a result, motif identification plays an important role in biological studies.

Motif prediction is usually the first stage in the process of identifying motifs. An extensive amount of research has been done on this topic over the past twenty years. In the literature, many approaches for motif prediction have been proposed. One of them is a combinatorial approach that has proven to be more accurate than the others. Even in this combinatorial approach, many variations can be found such as *Planted Motif Search (PMS)*, *Simple Motif Search (SMS)*, and *Edit-distance-based Motif Search (EMS)* (see e.g., [2]).

Among the combinatorial variations, the PMS Problem has been the most widely studied perhaps because it offers a higher level of accuracy in modeling the true motifs than the others. Motifs typically occur with mutations at binding sites. The binding sites are referred to as *instances* of a motif. A motif in this model is referred to as a 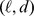-motif where 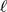 is its length and 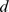 is the maximum number of mutations allowed for its instances. Given a set of sequences, the objective of the PMS problem is to find all the 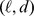-motifs in them. The formal definition of the PMS Problem is given in Section 0.1. An algorithm that solves the PMS problem is called a *PMS Algorithm*.

Owing to its importance, the PMS problem has been extensively studied in the past twenty years. Many PMS algorithms have been proposed in the literature. There are two kinds of PMS algorithms, namely, *exact* and *approximate*. An exact algorithm always finds all the 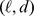-motifs present in the input sequences. An approximate algorithm may not find all the motifs. In this paper we only consider exact algorithms. The (exact variant of the) PMS problem has been shown to be NP-hard which means that there is unlikely to be a PMS algorithm that takes only polynomial time. As a result, all the existing exact PMS Algorithms take time that is exponential time in some of the parameters in the worst case. In practice, all known PMS Algorithms (both exact and approximate) are only able to find 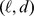-motifs for up to certain values of 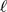 and 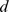. The most recent exact algorithms that have been proposed in the literature are Algorithm PMS6 due to [3], Algorithm PMS5 due to [4], Algorithm Pampa due to [5], Algorithm PMSPrune due to [6], Algorithm PMS3 due to [7], Algorithm Voting due to [8], and Algorithm RISSOTO due to [9]. Some earlier PMS algorithms are due to [10], [11], [12], [13], [14], [15], [16], and [17]. Among these known algorithms, Algorithm PMS6 is considered the fastest one and has been developed closely following the ideas of Algorithm PMS5.

Approximate PMS algorithms usually tend to be faster than exact PMS algorithms. Typically, approximate PMS algorithms employ heuristics such as local search, Gibbs sampling, expectation optimization, etc. Examples of approximate algorithms are Algorithm MEME due to [18], Algorithm PROJECTION due to [19], Algorithm GibbsDNA due to [20], Algorithm WINNOWER due to [21], and Algorithm RandomProjection due to [22]. Some other approximate PMS algorithms are Algorithm MULTIPROFILER due to [23], Algorithm PatternBranching due to [24], Algorithm ProfileBranching due to [24], and Algorithm CONSENSUS due to [25].

A generalized version of the PMS Problem, namely *Quorum Planted Motif Search* (qPMS) Problem, was first considered in [6]. The qPMS problem is to find all the motifs that have motif instances present in *q* out of the *n* input sequences. The qPMS problem captures the nature of motifs more precisely than the PMS problem does because, in practice, some motifs may not have motif instances in all of the input sequences. The qPMS problem is formally defined in Section 0.1. An algorithm that solves the qPMS problem is called a qPMS algorithm. qPMS algorithms can be used to find DNA motifs and protein motifs as well as transcription factor binding sites. The larger the values of 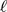 and *d* that a qPMS algorithm can handle, the more accurate will be the motifs it finds. So it is important to solve the qPMS problem instances with large values of 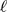 and *d*. However, solving the qPMS problem is a difficult task since it is even harder than the PMS problem. To the best of our knowledge, the currently best exact qPMS algorithm is Algorithm qPMSPrune due to [6] that can only solve instances up to 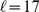 and 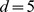 for 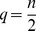, where *n* is the number of input sequences. In this paper, we propose a new algorithm named Algorithm qPMS7 that can solve larger instances. Also, qPMS7 is ten times as fast as qPMSPrune. In addition, when applied to the PMS problem, our algorithm is faster than the best PMS algorithm, i.e., Algorithm PMS6 due to [3].

## Methods

### 0.1 Problems Definition and Notations

**Definition 0.1** *A string 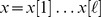 of length 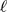 is called an 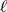-mer.*

**Definition 0.2** *Given two 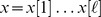 and 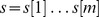 with 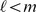, we use the notation 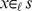 if x is a contiguous substring of s. In other words, 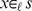 if there exists 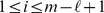 such that 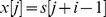 for every 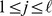. We also say that x is an 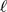-mer in s.*

**Definition 0.3** *Given two strings 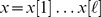 and 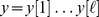 of equal length, the Hamming distance between 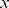 and 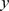, denoted by 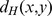, is the number of mismatches between them. In other words, 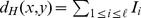, where 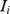 is the indicator at position 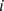. 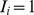 if 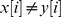, and 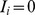 otherwise.*

**Definition 0.4** *Given two strings 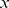 and 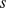 with 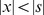, the Hamming distance between 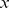 and 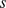, denoted by 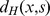, is 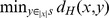.*

**Definition 0.5** *Given a set of 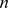 strings 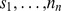 of length 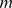 each, a string 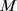 of length 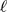 is called an 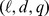-motif of the strings if there are at least 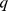 out of the 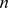 strings such that the Hamming distance between each one of them and 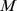 is no more than 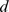. 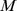 is called an 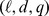-motif for short if the set of strings is clear.*

The definition of *Quorum Planted Motif Search (qPMS)* Problem is as follows. Given 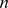 input strings 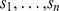 of length 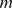 each, three integer parameters 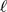, 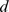 and 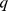, find all the 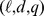-motifs of the input strings. The *Planted Motif Search (PMS)* problem is a special case of the qPMS problem when 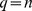. In this paper, we propose a fast algorithm for the qPMS problem.

### 0.2 The Existing Algorithm qPMSPrune

Algorithm qPMSPrune for the qPMS problem was proposed by [6]. For the sake of completeness, we will describe Algorithm qPMSPrune in this section briefly because our new algorithm is partially based on it. For more details on Algorithm qPMSPrune, the readers are referred to [6].

Algorithm qPMSPrune uses the 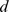-neighborhood concept defined as follows.

**Definition 0.6** *Given a string 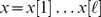, we define the 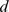
**-neighborhood** of 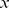, 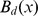, to be 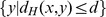.*

It is easy to see that 
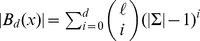
, where 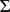 is the alphabet of interest. Notice that 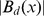 depends only on 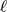, 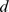 and 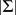. For this reason, we define 
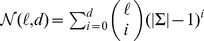
.

Algorithm qPMSPrune is based on the following observation. Any 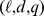-motif of the input strings must be in 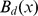 for some 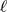-mer 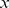 in some input string 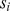 and also it must be a 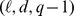-motif of the input strings excluding 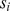. This observation can be rewritten formally as follows.

**Observation 0.1** *Let 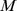 be any 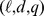-motif of the input strings 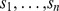. Then there exists an 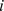 (with 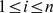) and a 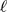-mer 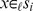 such that 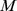 is in 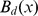 and 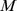 is a 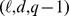-motif of the input strings excluding 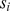.*

The above observation suggests the following algorithm. Compute 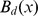 for every 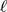-mer 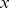 in each input string 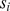 for 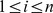. For each 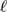-mer in the neighborhoods thus computed, check if it is a 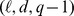-motif of the input strings excluding 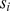. This simple algorithm can be improved further as shown in [6]. The key observation is that it is sufficient to consider each input string 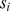 for 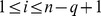:

**Observation 0.2** *Let 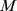 be any 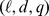-motif of the input strings 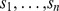. Then there exists an 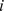 (with 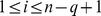) and a 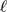-mer 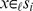 such that 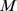 is in 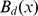 and 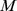 is a 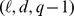-motif of the input strings excluding 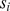.*

Algorithm qPMSPrune is based on the above observation. For any 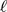-mer 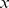, it represents 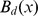 as a tree 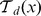 using the following rules.

1. Each node in 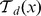 is a pair 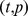 where 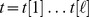 is an 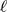-mer and 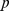 is an integer between 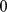 and 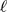 such that 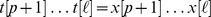. A node 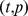 is referred to as a 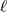-mer 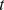 if 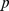 is clear.
2. Let 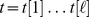 and 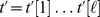. A node 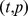 is the parent of a node 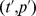 if and only if
  (a) 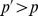.
  (b) 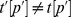 (From Rule 1, 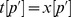).
  (c) 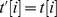 for any 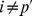.
3. The root of 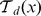 is 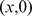.
4. The depth of 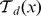 is 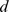.

**Figure 1:**
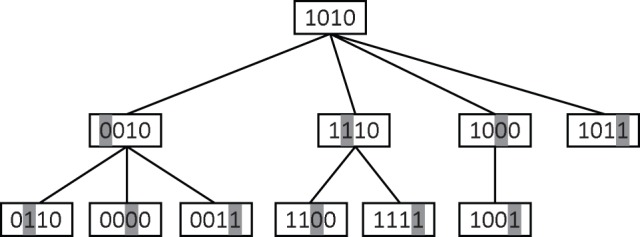
Traverse the tree in qPMSPrune. 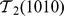
 with alphabet 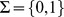. The value of 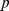 at each node is the location of its shaded letter. For example, 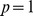 at node 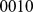, 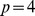 at node 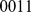.

For example, the tree 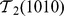 with alphabet 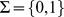 is illustrated in Figure 1.

Clearly, the following properties of 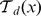 can be inferred directly from the rules.

1. Each node in 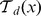 is uniquely associated with an 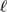-mer in 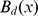 and vice versa.
2. If a node 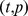 is a parent of a node 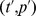, then 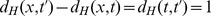. As a result, if a node 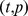 is at level 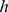, then 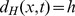.

The algorithm traverses the tree 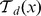 in a depth-first manner. At each node 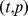, it computes 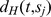 incrementally from its parent for 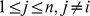. This operation can be done in 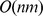 time by the incremental computation shown in [6]. Let 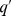 be the number of input strings 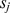 such that 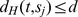. Obviously if 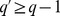 then 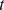 is a 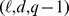-motif of the input strings excluding 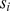. If this condition is satisfied, it outputs 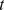 as a 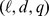-motif of the entire input strings.

Algorithm qPMSPrune prunes certain nodes (and their descendants) in 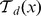 that cannot possibly be 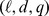-motifs. Under what conditions can we prune the node 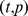
? Let 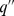 be the number of input strings 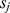 such that 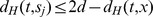. Observe that if 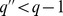 then none of the nodes in the subtree rooted at node 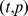 could be a 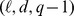-motif. This is because if there is a node 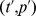 in the subtree which is a 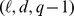-motif, then there are at least 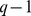 input strings 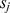 such that 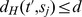. Consider such an input string 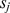. By the triangle inequality, 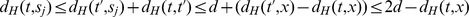. This inequality will infer that 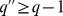. Therefore, if the condition 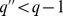 occurs, it can safely prune the subtree rooted at node 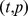 without missing any 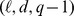-motif. The pseudo-code of Algorithm qPMSPrune is described as follows.

### Algorithm qPMSPrune

For each 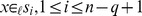 do:

Traverse the tree 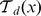 in a depth-first manner. At each node 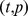, do the following steps.

- Incrementally compute 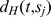 from its parent for 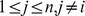.
- Let 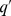 be the number of input strings 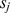 such that 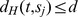. If 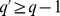, output 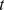.
- Let 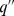 be the number of input strings 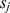 such that 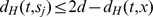. If 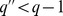, then prune the subtree rooted at node 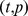. Otherwise, explore its children.

It is easy to see that the time and space complexities of Algorithm qPMSPrune are 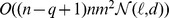 and 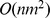, respectively.

### 0.3 A Computational Technique Improving upon Algorithm qPMSPrune

In this section, we propose a speedup technique to improve the runtime of Algorithm qPMSPrune. Specifically, the technique will reduce the time taken for computing Hamming distances 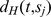 in step (1) of Algorithm qPMSPrune. Recall that the operation takes at least 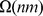 time in Algorithm qPMSPrune because it considers every 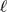-mer in each input string 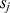. We observe that some 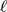-mers can be ignored without changing the result since we notice that we just need to count 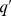 and 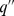. Any 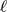-mer 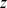 in 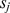 can be ignored, as far as a node 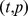 in the tree 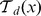 is concerned, if 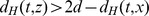. The reason for this will be given in the next paragraph. Based on this observation, the technique is implemented as follows. At each node 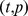, we store a list of surviving 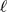-mers for each input string 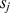. It is sufficient to store the positions of the 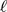-mers in the input strings. If the list of surviving 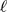-mers of 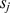 is empty, then we set 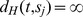. In terms of the incremental distance computation, only the surviving 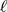-mers are considered. The runtime of the operation now depends on the sizes of the lists of surviving 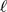-mers.

The reason for ignoring any 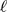-mer 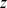 in 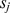, as far as a node 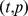 in the tree 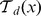 is concerned, if 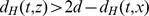 is as follows. If this condition occurs, then for any node 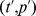 in the subtree rooted at node 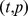 we have: 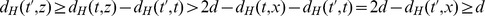. Therefore, ignoring 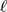-mer 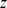 at any node 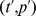 in the subtree rooted at node 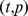 will not change its 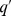. The value of 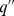 at node node 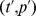 may become smaller as a result of ignoring the 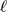-mer 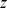. However, the pruning condition based on 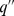 in step (3) in the pseudo-code still holds.

Another way to view the ignoring condition is as follows. Consider a node 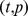 in the tree 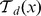 and an 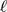-mer 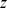 in the input string 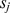. Let us separate each of 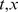 and 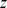 into two parts based on 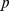, namely, 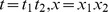 and 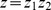 where 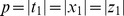 and 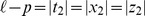. Notice that 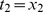. Then the inequality 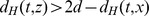 is equivalent to 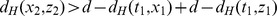. In other words, 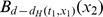 and 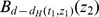 are disjoint. Notice that this condition is independent of 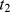. This view helps us in designing our best algorithm qPMS7 which is described in Section 0.4.

The speedup technique reduces the runtime of Algorithm qPMSPrune drastically because the deeper a node is, the smaller will be the size of its list of surviving 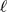-mers. Note that the number of nodes at a depth of 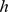 from the root will be exponential in 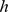. In practice, the runtime of Algorithm qPMSPrune is improved by a factor of around 5 when this technique is used (see Table 1 and Table 2). However, it does not change the worst case time complexity of Algorithm qPMSPrune, theoretically.

**Table 1:** Time comparison of different algorithms on the challenging instances of DNA sequences for the special case - PMS Problem.

Algorithm	(13,4)	(15,5)	(17,6)	(19,7)	(21,8)	(23,9)
qPMS7	47 s	2.6 m	11 m	0.9 h	4.3 h	24 h
PMS6	67 s	3.2 m	14 m	1.16 h	5.8 h	–
PMS5	117 s	4.8 m	21.7 m	1.7 h	9.7 h	54 h
qPMSPruneI	17 s	2.6 m	22.6 m	3.4 h	29 h	–
Pampa	35 s	6 m	40 m	4.8 h	–	–
qPMSPrune	45 s	10.2 m	78.7 m	15.2 h	–	–
Voting	104 s	21.6 m	–	–	–	–
RISOTTO	772 s	106 m	–	–	–	–

The alphabet 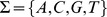, 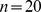, 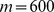, and 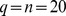.

### 0.4 Our Best Algorithm qPMS7

In this section, we propose a fast algorithm called qPMS7 for the qPMS problem. Algorithm qPMS7 is a generalized version of Algorithm qPMSPrune combined with the core idea of Algorithm PMS5 which was introduced in [4].

Recall that Algorithm qPMSPrune considers one 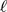-mer 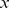 in a specific input string 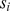 at a time. Algorithm qPMS7 extends Algorithm qPMSrune by considering two 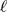-mers 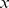 and 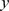 in two different input strings 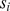 and 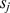. An observation similar to that of Algorithm qPMSPrune can be obtained as follows.

**Observation 0.3** *Let 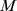 be any 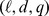-motif of the input strings 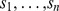. Then there exist 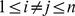 and 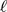-mer 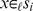 and 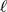-mer 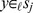 such that 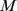 is in 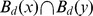 and 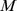 is a 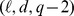-motif of the input strings excluding 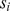 and 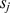.*

**Table 2:** Time comparison of different algorithms on the challenging instances of DNA sequences for the generalized case - qPMS Problem.

Algorithm	(13,3)	(15,4)	(17,5)	(19,6)	(21,7)
qPMS7	34 s	2.4 m	16 m	1.8 h	11.6 h
qPMSPruneI	14 s	2 m	21 m	3.9 h	–
qPMSPrune	32 s	9 m	2.6 h	–	–

The alphabet 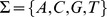, 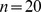, 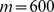, and 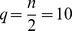.

Using an argument similar to the one in [6], we infer that it is enough to consider every pair of input strings 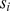 and 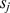 with 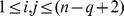. As a result, the above observation gets strengthened as follows.

**Observation 0.4** *Let 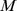 be any 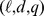-motif of the input strings 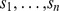. Then there exist 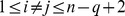 and 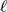-mer 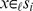 and 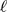-mer 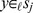 such that 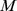 is in 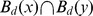 and 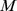 is a 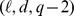-motif of the input strings excluding 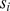 and 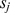.*

Like Algorithm qPMSPrune, Algorithm qPMS7 uses a routine that finds all of the motifs 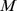 such that 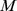 is in 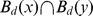 and is a 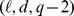-motif of the input strings excluding 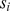 and 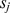. Recall that Algorithm qPMSPrune explores 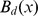 by traversing the tree 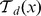. In Algorithm qPMS7, we also explore 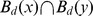 by traversing an acyclic graph, denoted as 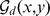, with similar construction rules. The rules for constructing 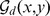 are given below.

1. Each node in 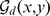 is a pair 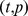 where 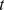 is an 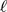-mer and 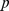 is an integer between 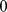 and 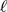. A node 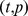 is referred to as 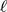-mer 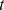 if 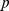 is clear. Let 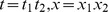 and 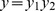 where 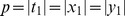 and 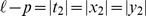. Node 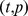 must satisfy the following constraints:
  (a) 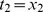 if 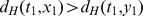, otherwise, 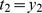.
  (b) 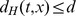 and 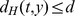.
2. Let 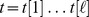 and 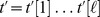. There is an arc from a node 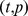 to a node 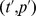 if and only if
  (a) 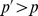.
  (b) 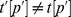.
  (c) 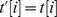 for any 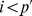.

**Figure 2:**
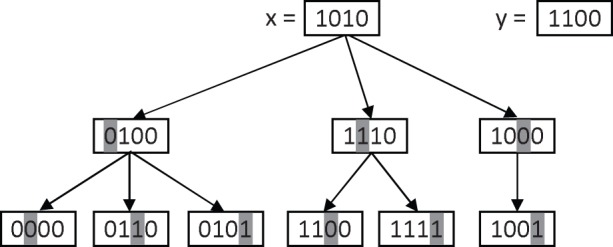
Traverse the graph in qPMS7. Visited nodes in 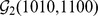 in a depth-first manner when the starting node is 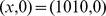. In this example, 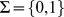. The value of 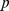 at each node is the location of its shaded letter. For example, 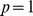 at node 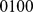.

It is not hard to see that if we traverse the graph 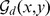 in a depth-first manner starting from node 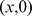, then all the 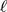-mers in 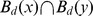 will be visited. For example, Figure 2 illustrates the visited nodes in the graph 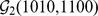 in a depth-first manner starting from node 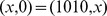 where the alphabet 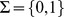.

Algorithm qPMS7 traverses the graph 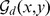 in a depth-first manner with the starting node 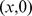. During the traversal, at each node 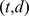 it computes 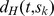 incrementally from its parent for 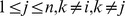. With the same method as the one in Algorithm qPMSPrune, we can achieve this task in 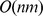 time. Also, it is easy to see that if 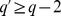 then 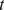 is a 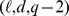-motif of the input strings excluding 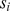 and 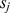, where 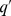 is the number of input strings 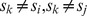 such that 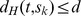. If this is the case, it outputs 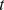 as a 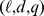-motif of the entire input strings.

Algorithm qPMS7 also uses a similar pruning strategy to that of Algorithm qPMSRune and the speedup technique discussed in Section 0.3. In this case, the speedup technique ignores some 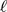-mers in 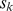 when computing 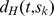 at each node 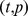 during the traversal of the graph 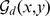. The ignoring condition of an 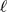-mer 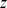 in 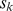 for this case resembles that in Section 0.3. Let 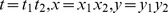 and 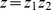 where 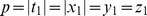. It is not hard to see that 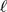-mer 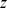 can be safely ignored if 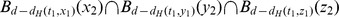 is empty. Checking for this condition can be done in 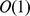 time using the incremental computation shown in [4]. During the traversal of the graph, at each node 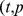) we also store a list of surviving 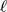-mers for each input string 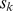. At node 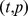, if the list of surviving 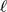-mers of an input string is empty, then the input string will contribute nothing to any descendant node of 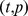 in order for that descendant to be a 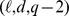-motif. Therefore, the pruning condition is 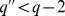 where 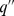 is the number of input strings whose lists of surviving 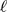-mers are not empty. The following pseudo-code describes Algorithm qPMS7.

### Algorithm qPMS7

1. For each 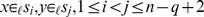 do:

(a) Traverse the graph 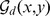 in a depth-first manner starting from node 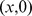. At each node 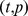, do the following steps.

- Incrementally compute 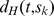 from its parent for 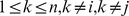.
- Let 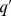 be the number of input strings 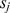 such that 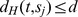. If 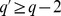, output 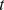.
- Let 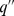 be the number of input strings whose lists of surviving 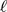-mers are not empty. If 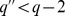, then backtrack. Otherwise, explore its children.

Theoretically, the time and space complexities of Algorithm qPMS7 are 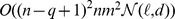 and 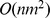, respectively. In the worst case scenario, the runtime of Algorithm qPMS7 is worse than that of Algorithm qPMSPrune by a factor of 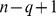. However, Algorithm qPMS7 is much faster than Algorithm qPMSPrune in practice, as shown in Section 0.5.

Algorithm qPMS7 also employs the following observation which has been used in many prior works such as [6] and [26]. Let 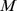 be any 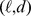 motif in inputs strings 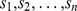. Let 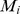 be an instance of 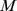 in 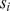 (for 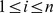). Then the Hamming distance between 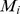 and 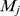 is 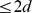 for any 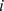 and 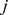 (with 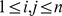). In other words, if 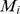 is any 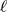-mer in some 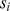, then it could possibly be an instance of 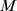 only if there are at least 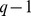 out of 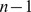 sequences 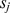
‘s, 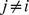, that have an 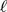-mer 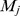 such that the Hamming distance between 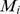 and 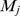 is 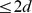. This observation can be utilized to preprocess the input strings so that for any input string only those 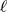-mers that satisfy the above condition are kept (and the other 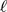-mers are ignored from further processing).

### 0.5 Transcription Factor Binding Sites Discovery

In this section we will discuss how to use a qPMS Algorithm, e.g., Algorithm qPMS7, to discover transcription factor-binding sites. Given a set of DNA strings that likely contains transcription factor-binding sites, we propose a general framework to find them. The framework consists of two phases. The first phase will select a set of motifs by repeatedly calling the qPMS Algorithm on different values of 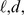 and 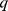. The second phase will use a scoring function to eliminate some of the motifs returned in the first phase, and then identify the transcription factor-binding sites based on the surviving motifs.

In the first phase we employ different values, ranging between 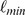 and 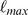, for the length 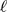 of motifs, where 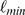 and 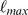 are user-specified parameters. For each value of 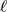, we let 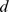 range from 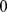 to 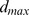, where 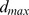 is another user-specified parameter, and call the best qPMS algorithm (let it be Algorithm 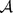) to find 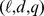-motifs. In this process, if some 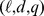-motif(s) are found, we add them to the set of motifs. The pseudo-code of the first phase follows.

**Phase I:** selecting candidate motifs

**Input:** a set of strings

**Parameters:** 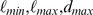 and 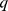


**Output:** a set of 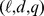-motifs 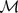


1: 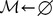


2: **for** 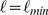 to 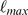 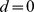 to **do**

3: **for** *d* = 0 to 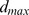 **do**

4: Run the fastest qPMS Algorithm 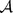 to find 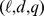-motifs of the input strings

5: **if** algorithm 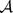 takes too long **then**

6: Terminate algorithm 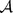


7: **break** the for loop of 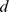


8: **end if**

9: Let 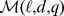 be the set of 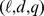-motifs returned by algorithm 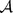


10: **if** 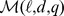 is **NOT** empty **then**

11: 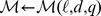


12: **break** the for loop of 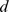


13: **end if**

14: **end for**

15: **end for**

In the second phase, we sort the 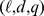-motifs according to their scores and pick the top 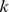 motifs, where 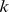 is a user-specified parameter. For each picked 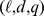-motif 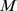 and each input string 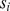, transcription binding sites in 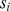 are identified as follows. We consider every 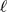-mer 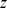 in 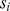 and output the location of 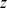 in 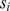 as a transcription binding site if 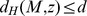. The following pseudo-code describes the second phase.

**Phase II:** identifying transcription factor binding sites

**Input:** a set of strings and a set of 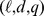-motifs 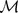


**Parameters:** a scoring function and 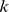


**Output:** a set of binding sites on the input strings

1: Sort 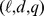-motifs in 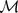 according to the scoring function

2: Pick the top 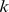 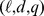-motifs in 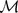 after sorting

3: **for** each picked 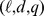-motif 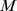 **do**

4: **for** each input string 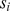 **do**

5: Identify all the 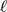-mers 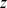 in 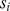 such that 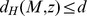


6: Output the location of each such 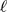-mer 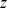 in 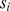 as a transcription factor binding site

7: **end for**

8: **end for**

**Table 3:** Time comparison of different algorithms on the challenging instances of protein sequences for the special case - PMS Problem.

Algorithm	(11,5)	(13,6)	(15,7)	(17,8)	(19,9)
qPMS7	1 m	1.4 m	1.9 m	6.8 m	7.5 m
qPMSPruneI	4.5 m	21 m	2.4 m	17 h	–
qPMSPrune	12 m	104 m	16 h	–	–

The alphabet size 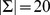, 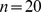, 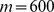, and 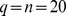.

The accuracy of the framework in discovering transcription factor-binding sites heavily depends on two factors: the qPMS Algorithm and the scoring function. Of course, the faster the qPMS Algorithm is, the more accurate will be the results it provides. Designing fast qPMS algorithms is our main focus because it is a difficult task. On the other hand, the choice of the scoring function is also critical. In general, the scoring function should measure the biological significance of a candidate motif possibly via a probabilistic model. As a rule of thumb, the smaller the probability that a motif appears (by random chance) is, the more likely will it be to be biologically significant. In addition, the impact of the scoring function on the accuracy also depends on the size of the list of candidate motifs 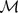. The larger the size is, the more will be the scoring function’s impact. For example, the scoring function called “sequence specificity” is usually used. It is defined to be 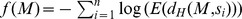 where 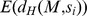 is the expected number of times a motif appears in string 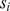 with up to 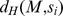 mismatches [6].

**Table 4:** Time comparison of different algorithms on the challenging instances of protein sequences for the generalized case - qPMS Problem.

Algorithm	(11,4)	(13,5)	(15,6)	(17,7)	(19,8)
qPMS7	27 s	3 m	18 m	3.8 h	11 h
qPMSPruneI	62 s	16 m	3.7 h	–	–
qPMSPrune	181 s	113 m	29 h	–	–

The alphabet size 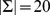, 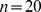, 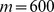, and 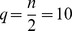.

## Results

In this section we evaluate the performance of Algorithm qPMS7 on simulated as well as real data. With simulated data, we compare its runtime with that of other existing algorithms. With real data, we measure the accuracy of qPMS7 in detecting real motifs. Of course, the larger the values of 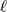 and 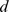 that an algorithm can solve, the more accurate will be the results it yields because it covers a larger search space of motifs.

**Table 5:** Results on real datasets.

Data	Predicted Motifs	Known Motifs	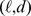
1	CCTCAGCCCC	CCTCAGCCCC	(10,2)
2	ATTTCGTGGCA	ATTTCnnGCCA	(13,2)
3	CCATATTAGGACATCT	CCATATTAGGACATCT	(16,3)
4	TTTCCCATTAAGGAAA	TTTCCCnnTnAGGAAA	(16,3)

Data 1: Preproinsulin; Data 2: DHFR; Data 3: c-fos; Data 4: Yeast ECB. Parameter 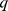 is set to 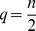.

### 0.6 Experiments on Simulated Data

We compared the runtime of Algorithm qPMS7 with other well-known algorithms such as Algorithm qPMSPrune of [6], Algorithm PMS6 of [3], Algorithm PMS5 of [4], Algorithm Pampa of [5], Algorithm Voting of [8], and Algorithm RISSOTO of [9]. Recall that among these algorithms, only Algorithm qPMSPrune deals with the qPMS problem. The rest of the algorithms deal with the simpler version, i.e., the PMS problem. The improved Algorithm qPMSPrune in Section 0.3 is named qPMSPruneI. To evaluate the performance of algorithms, we usually test them on challenging and hard instances of the problem. All of these algorithms have been run on the same machine running Windows XP Operating System with a Dual Core Pentium 2.4GHz CPU and 3GB RAM. The experimental results below show that Algorithm qPMS7 is better than any other algorithm.

**Table 6:** Results on real datasets for transcription factor-binding sites discovery.

Data	Predicted Motifs	Matched Binding Sites
mus05r	AGAGGAAAAAAAAAAGGAG	*s*_1_: GGAAAAACAAAGGTAATG
mus07r	CTGCCCACCCTCTGCAACCC	*s*_4_: CCCAACACCTGCTGCCTGAGCC
mus11r	AGGGCGGGGGGCGGAGCG	*s*_2_: GCCGCCGGGGTGGGGCTGAG
		*s*_3_: GGGGGGGGGGGCGGGGC
		*s*_4_: GTGGGGGCGGGGCCTT
		*s*_9_: GAACAGGAAGTGAGGCGG
hm03r	AAAAGAAAAAAAAATAAACAA	*s*_1_: TCAAGCAAAAAAAATAAATAAATACCTATGCAA
		*s*_2_: ACAAGCAAACAAAATAAATATCTGTGCAATAT
		*s*_3_: TATGAGCAAACAAAATAAATACCTGTGCAA
hm08r	CGTGCAGTCCCCTTCAT	*s*_10_: TATGGTCATGACGTCTGACAGAGC
hm19r	CCCTTCCACCACCCACAG	*s*_2_: CACTTTTAGCTCCTCCCCCCA
hm26r	CCCCCCGCCTCCCGCTCCC	*s*_3_: CCCCGCCTCAGGCTCCCGGGG
		*s*_7_: CTCAGCCTGCCCCTCCCAGGGATTAAG
		*s*_8_: GCGCCGAGGCGTCCCCGAGGCGC

The datasets are from mouse (resp. human) if their names start with “mus” (resp. “hm”).

#### 0.6.1 DNA sequences

Following [21] and [6], the set of input strings of a challenging instance is typically generated as follows. Each input string is a random DNA string drawn according to the i.i.d model. A random 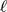-mer 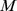 is chosen as a 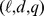-motif and mutations of this 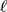-mer 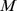 are planted in 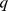 out of the 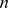 input strings at random positions. The Hamming distance between 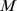 and any of these mutations is at most 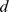. The number of input strings 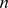 and the length of each of them 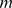 are chosen to be 20 and 600, respectively.

In the case of the PMS problem, 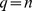. The pairs 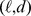 corresponding to challenging instances are 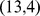, 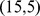, 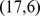, 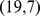, 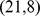, 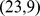, 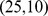, and so on. To the best of our knowledge, there has not been any algorithm that can solve the challenging instance 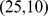. Therefore, Table 1 reports the runtime of the algorithms on the challenging instances up to 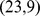. Algorithms qPMS7, PMS6 and PMS5 can solve any of these challenging instances. In Table 1, the letter ‘–’ indicates that the corresponding algorithm either takes too long or uses too much memory on the corresponding challenging instance.

Following [6] and [4], we have tested the qPMS algorithms on the most difficult case of 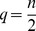. The challenging instances for this case are identified as 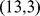, 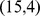, 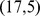, 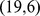, 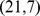, 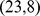, 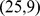, and so on. Since among the existing algorithms, only Algorithm qPMSPrune deals with the qPMS problem, we compare Algorithm qPMS7 to it. Algorithm qPMS7 can solve any challenging instance up to 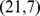. Table 2 shows the results for these challenging instances.

#### 0.6.2 Protein sequences

We have also tested the algorithms on synthetic protein sequences. These sequences have been generated in a manner similar to the generation of DNA strings as explained in Section 0.6.1. The number of the protein strings in each testing dataset 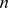 and the length of each protein string 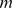 are chosen to be the same: 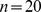 and 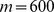. For the case of 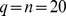, the 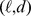 pairs that correspond to challenging instances are 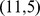, 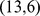, 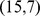, 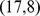, 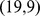, and so on. For the case of 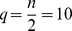, the challenging instances are 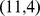, 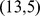, 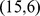, 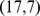, 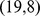, and so on.

Since none of the exact algorithms reported in the literature deals with the qPMS problem for protein sequences, we restrict our comparison to the algorithms qPMSPrune, qPMSPruneI, and qPMS7. Table 3 and Table 4 show the runtimes of these algorithms for the two cases 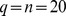 and 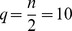, respectively. As the results show, Algorithm qPMS7 outperforms Algorithms qPMSPruneI and qPMSPrune on all the cases.

### 0.7 Experiments on Real Data

#### 0.7.1 Finding real DNA motifs

We tested Algorithm qPMS7 on the real datasets discussed in [27] which is commonly used to measure the accuracy of the existing algorithms (see e.g., [27], [19], and [7]). Each of the datasets is a collection of DNA orthologous sequences from many organisms. These real datasets are substantially different from the simulated data because they contain known transcription regulatory elements, i.e, known motifs. Algorithm qPMS7 was able to identify these known motifs for appropriate values of the parameters 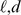 and 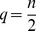. We report these motifs in Table 5. However, we should mention that our results are similar to those published in [7], [6] as well as other papers.

#### 0.7.2 Detecting transcription factor-binding sites

We have also tested our algorithms on the biological datasets described in [28]. In this collection there are several datasets. Some strings of each of these datasets contain known transcription factor-binding sites of different lengths and the others do not. Therefore, in order to test these real datasets we rely on the framework for transcription factor-binding sites discovery described in Section 0.5. Recall that this framework needs a qPMS algorithm and a scoring function. Since Algorithm qPMS7 is currently the fastest, we employ it in this framework. Regarding the scoring function, we use the function called “sequence specificity” which is also the one used in [6], which basically is defined to be 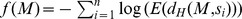 where 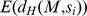 is the expected number of times a motif appears in string 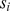 with up to 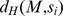 mismatches, assuming the i.i.d model. To complete the tests, we need to choose the parameters of the framework 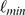, 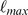, 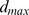, 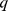, and 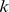. We set 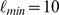, 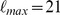, 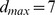, 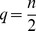, and 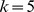. With this setting, we obtain good results like those in [28], [6], and [4] with many transcription factor-binding sites predicted correctly. Table 6 reports some of these correctly predicted binding sites together with the predicted motifs.

## Discussion

In this paper we have presented Algorithm qPMS7 for the qPMS problem and tested it on DNA as well as protein sequences. Experimental results indicate that Algorithm qPMS7 is faster than other existing algorithms, especially for large values of 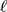 and 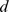. Since Algorithm qPMS7 is a search-based algorithm, it uses a small amount of memory. This feature of Algorithm qPMS7 is a major advantage compared to other algorithms such as RISOTTO, Voting, PMS5, and PMS6 which require a large amount of memory when solving instances with large values of 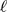 and 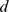. Another advantage of Algorithm qPMS7 over these algorithms is that they cannot deal with the qPMS problem and in particular they only handle the PMS problem.

Algorithm qPMS7 is the result of a combination of an extension of Algorithm qPMSPrune and the core idea of algorithm PMS5. In Algorithm qPMSPrune, a “pivot” 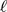-mer is used. In Algorithm qPMS7, we extended this idea by considering two pivot 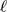-mers. This idea can be further generalized by considering more than two pivot 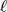-mers,

In this paper we have also proposed a framework for transcription factor-binding sites discovery. It should be mentioned that our framework together with Algorithm qPMS7 is currently deployed in our online tools at http://pms.engr.uconn.edu or at http://motifsearch.com. We will be very happy to receive any comments and feedback from users.
